# Detection of *Talaromyces marneffei* from Fresh Tissue of an Inhalational Murine Pulmonary Model Using Nested PCR

**DOI:** 10.1371/journal.pone.0149634

**Published:** 2016-02-17

**Authors:** Yinghui Liu, Xiaowen Huang, Xiuwen Yi, Ya He, Eleftherios Mylonakis, Liyan Xi

**Affiliations:** 1 Department of Dermatology, Sun Yat-Sen Memorial Hospital, Sun Yat-Sen University, Guangzhou, China; 2 Department of Dermatology, General Hospital of Guangzhou Military Command of PLA, Guangzhou, China; 3 Division of Infectious Disease, Rhode Island Hospital, Waren Alpert Medical School of Brown University, Providence, Rhode Island, United States of America; Rutgers University, UNITED STATES

## Abstract

Penicilliosis marneffei, often consecutive to the aspiration of *Talaromyces marneffei* (*Penicillium marneffei*), continues to be one of the significant causes of morbidity and mortality in immunocompromised patients in endemic regions such as Southeast Asia. Improving the accuracy of diagnosing this disease would aid in reducing the mortality of associated infections. In this study, we developed a stable and reproducible murine pulmonary model that mimics human penicilliosis marneffei using a nebulizer to deliver *Talaromyces marneffei* (SUMS0152) conidia to the lungs of BALB/c nude mice housed in exposure chamber. Using this model, we further revealed that nested PCR was sensitive and specific for detecting *Talaromyces marneffei* in bronchoalveolar lavage fluid and fresh tissues. This inhalation model may provide a more representative analysis tool for studying the development of penicilliosis marneffei, in addition to revealing that nested PCR has a predictive value in reflecting pulmonary infection.

## Introduction

*Talaromyces marneffei* (*T*. *marneffei*) which former name was *Penicillium marneffei* [1], is a thermally dimorphic fungus that causes lethal penicilliosis marneffei [1, 2]. The last four decades have witnessed an increasing incidence of infection since the first case was reported in 1984 [3, 4]. Despite advancements in medical mycology, mortality of penicilliosis marneffei remains high, about 51% in untreated patients and 24.3% in treated patients [4]. Hence, further study focused on penicilliosis marneffei is urgently needed to reduce mortality associated with this disease.

To date, several murine models have been developed to study this disease. N. Kudeken et al. infected mice by intratracheal instillation of *T*. *marneffei* to define the host immune response against this pathogen [5, 6]. Sun et al. used a systemic murine model which relies on the injection of a suspension of *T*. *marneffei* yeast cells into the lateral tail vein of mice to assess the virulence of different strains [7]. Though these studies provided some new insights, the methodologies are time consuming or don’t represent typical human exposures routes. A reproducible and simple-to-operate animal model that can precisely mimic human pulmonary penicilliosis marneffei is critical for developing new methods to diagnose and treat this disease.

Molecular biology methods are helpful for early and rapid diagnosis of infectious disease. With regard to *T*. *marneffei*, various PCR-based methods have been proposed [8–12]. For its sensitive and rapid identification, nested PCR was employed. However, the resource of sampling DNA is always limited to lab cultures, tissue embedded samples [10] or whole blood samples [12]. Up to now, nested PCR has not been evaluated in fresh tissues. Hoping to spread its application in a clinical setting, we constructed a murine infection model in this study by utilizing an inhalation chamber. We then evaluated the performance of a nested PCR assay in identifying *T*. *marneffei* in fresh tissue samples and bronchoalveolar lavage fluid (BALF).

## Materials and Methods

### Strains and Mice

*T*. *marneffei* strain (SUMS0152) was used for all experiments [13, 14]. It was grown on potato dextrose agar (PDA) (Becton, Dickinson and Company, USA) at 25°C for 2 weeks, conidia were then collected by flooding the culture surface with phosphate buffer solution (PBS). The resulting fungal suspensions were adjusted to the required concentrations using a hemocytometer [12].

Specific pathogen-free female BALB/c nude mice weighting 20 to 22 g, (the experiment animal center of Sun Yat-sen University, Guangzhou), were acclimated for one week prior to exposures. Mice were housed in individual ventilated cage with irradiated food and sterile water available and their body weight were monitored everyday. Mice were euthanized by inhaling gradually increasing concentrations carbon dioxide for 5 min when one of the following symptoms showed: inability to ambulate, inability to access food or water, emaciation (the main sign of emaciation is more than 20% weight loss from start along with hunched posture). All procedures involving mice were supervised and approved by the Institutional Animal Care and Use Committee (IACUC) and Ethics Committee of Sun Yat-Sen University. The permit number for the animal ethics is 2013–1101.

### Aerosol exposure system

**Fig 1:**
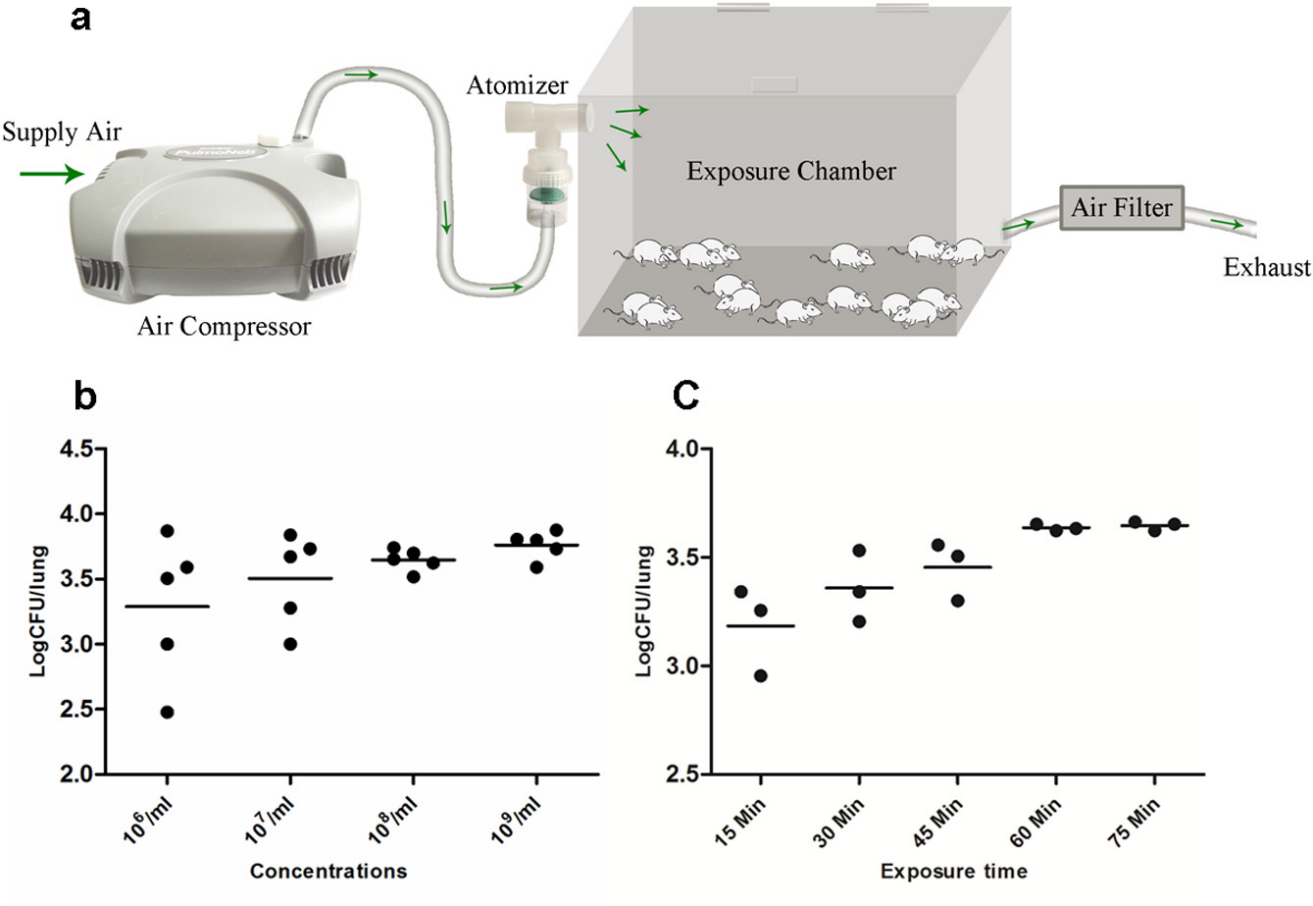
Preliminary experiments of aerosol exposure system. Illustration shows the inhalation exposure system. Air is directed into the compressed air-driven nebulizer, and then directed into a multi-animal exposure chamber. After passing through the exposure chamber the air is filtered before being sent into the exhaust system (a). Fungal burdens in lung of each mouse after exposure to conidia suspensions of various concentrations for 60 min (b) and a suspension containing 10^8^/mL for different times (c).

An aerosol exposure apparatus were assembled according to the description of Donald C. Sheppard (Fig 1A) [15]. Mice were introduced to the exposure chamber (Yuyan instruments company, Shanghai, China) via a hinged door on the top of chamber. Conidia suspensions were aerosolized using a Nebulizer (DeVilbiss, Shanghai, China) driven by compressed air. The generated aerosol was connected to the exposure chamber, which was connected to an air filter (Yuyan instruments company, Shanghai, China) though another tube to prevent spores from contaminating the environment. The entire apparatus can be completely sealed.

### Determination of exposure time and inoculum concentration

To select the appropriate inoculum concentration, mice received aerosols generated from conidial suspensions with different concentration for a standard 60 min, then sacrificed immediately after exposure. The lungs, livers, spleens and kidneys were removed and homogenized for colony-forming unit (CFU) determinations. To determine the optimal exposure time, mice were exposed to a selected spore suspension for different exposure time of 15 min, 30 min, 45 min, 60 min or 75 min, then the same procedure was performed for CFU analysis.

### The infection process

Forty-four mice received aerosols generated from 8 mL of a suspension containing 10^8^ conidia/ml for a fixed exposure time of 60 min for complete and uniform exposure of all the mice. Twenty mice received aerosols generated from PBS as control. Twenty mice were used for survival assay. The rest were randomly selected at select time points for further study.

### Collection of BALF

BALF was collected as described previously [16]. Briefly, mice were sacrificed and a small incision was made at the neck to expose trachea. A SURFLO catheter was cannulated into the trachea under the larynx. Sterile PBS were flushed through the animal lung, and then transferred to a 15 mL centrifuge tube (Biofil Guangzhou China). This process was repeated until 4 mL of BALF was collected. BALF was centrifuged at 12,000 rpm for 10 min. Remaining sediments were resuspended with PBS with the final volume of 0.5 mL.

### Histology

On days 0, 3, 7, 15, 21, and 30, four mice were randomly selected. Organ samples were removed from sacrificed animals. For histopathological study, half of each organ was fixed in 4% neutral buffered formalin, processed, and embedded in paraffin. Tissue sections were stained with hematoxylin and eosin (H&E) or schiff periodic acid shiff (PAS) stain. The presence or absence of yeast cells, their morphology and location (intracellular or extracellular), and the host inflammatory response were recorded.

### Colony forming units (CFU) determination

For determination of CFU, primary homogenates of each half of organ were prepared by adding 1 ml sterile saline per gram of tissue. Serial 10-fold dilutions of primary homogenates were plated in triplicate on PDA at 25°C for 3 days before colonies were counted.

### DNA extraction

About 0.5 mL of primary homogenate and 0.5 mL BLA was used for DNA extraction with the InstaGene Matrix (Bio-Rad, Hercules, CA) as described by the manufacturer’ s instructions. The prepared DNA samples were stored at -20°C until use.

### Nested PCR

Nested PCR assay were performed as described previously [8,13]. Briefly, the out primers were RRF1: 5’ATCTAAATCCCTTAACGAGGAACA3’ and RRH1: 5’CCGTCAATTTCTTTAAGTTTCAGCCTT3’. The inner primers were Pm1: 5’ATGGGCCTTTCTTTCTGGG3’ and Pm2: 5’GCGGGTCATCATAGAAACC3’ (synthesized by Shenggong company, Shanghai, China). In the primary PCR, 2.5μL template DNA were added to a 25 μL reaction system containing 12.5μL Premix Taq DNA polymerase (Takara, Dalian, China), and 1μM of out primers and 8μL of RNase-free water. The parameter for PCR reaction was: 95°C for 5 min; 35 cycles of 95°C for 30 s, 55°C for 30 s, 72°C for 2 min and final extension at 72°C for 10 min. One micro liter of the primary PCR product was subjected to second PCR amplification. The parameters of amplification in nested PCR were the same as described in the primary PCR except that the annealing temperature of 68°C and specific inner primer. Five microliters of nested PCR amplification products were analyzed by electrophoresis (Bio-Rad, California, USA) and 1% agarose gels (Invitrogen, USA). Expected sizes of the nested PCR products were about 400-bp.

DNA from selected fungal species and mice were used to test specificity of the nested PCR. The sensitivity of nested PCR was determined by comparing it with single PCR assay carried out under the same condition described above with primer pair Pm1 and Pm2. Briefly, 1μl of the DNA solution from BALF sample (8.4×10^9^ fg/μl) was used as a template for PCR and serially diluted 10-fold in RNase-free water. The amplification products were analyzed by electrophoresis.

### Statistical analysis

Graphs were plotted by using GraphPad Prism 5 software (GraphPad Software, La Jolla, CA). Survival data were analyzed by means of log-rank comparisons of Kaplan–Meier survival curves. Coefficient of variation was used to access the variation of CFU of lung within each group. Fungal burden were statistically processed by t-test. Statistical analyses were considered significant when *P* value of less than 0.05 was produced.

## Results

### Characterization of the fungal aerosol exposure system and titration of infectious inoculum

We adapted an inhalation apparatus to infect mice via respiration (Fig 1A), and evaluated it by exposing mice to suspensions of different concentrations for a fixed time of 60 min [15]. We found that nebulizing 8 mL of the suspension containing 10^8^/mL conidia for 60 min delivered a stable and reproducible inoculum in mice, and reached to 5×10^3^ CFU in lung tissues. Lower concentration produced variable levels of inoculums (P = 0.0032 for 10^6^/mL, P = 0.0043 for 10^7^/mL). Higher conidia concentration (P = 0.6702) and longer exposure time (P = 0.4516) produce similar lung tissue CFU as conidial concentration of 10^8^/mL for 60 min. (Fig 1B and 1C).

### Survival assay and analysis of fungal burden within infected mice

**Fig 2:**
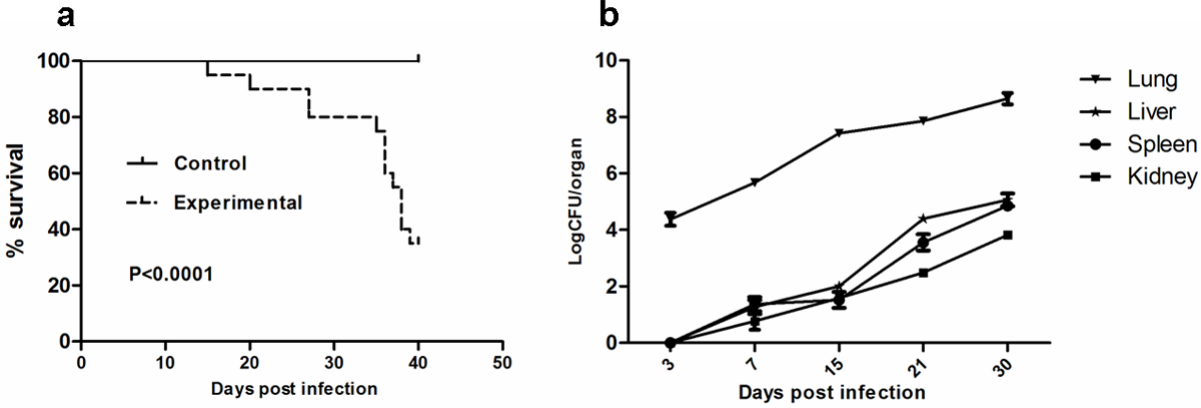
Survival and fungal burdens after inhalational infection with *T*. *marneffei*. Survival curves (a) and fungal loads in lung, spleen, liver and kidney at selected time post infection (b) of BALB/c nude mice received 5×10^8^ conidia of *T*. *marneffei* strain 152.

**Fig 3:**
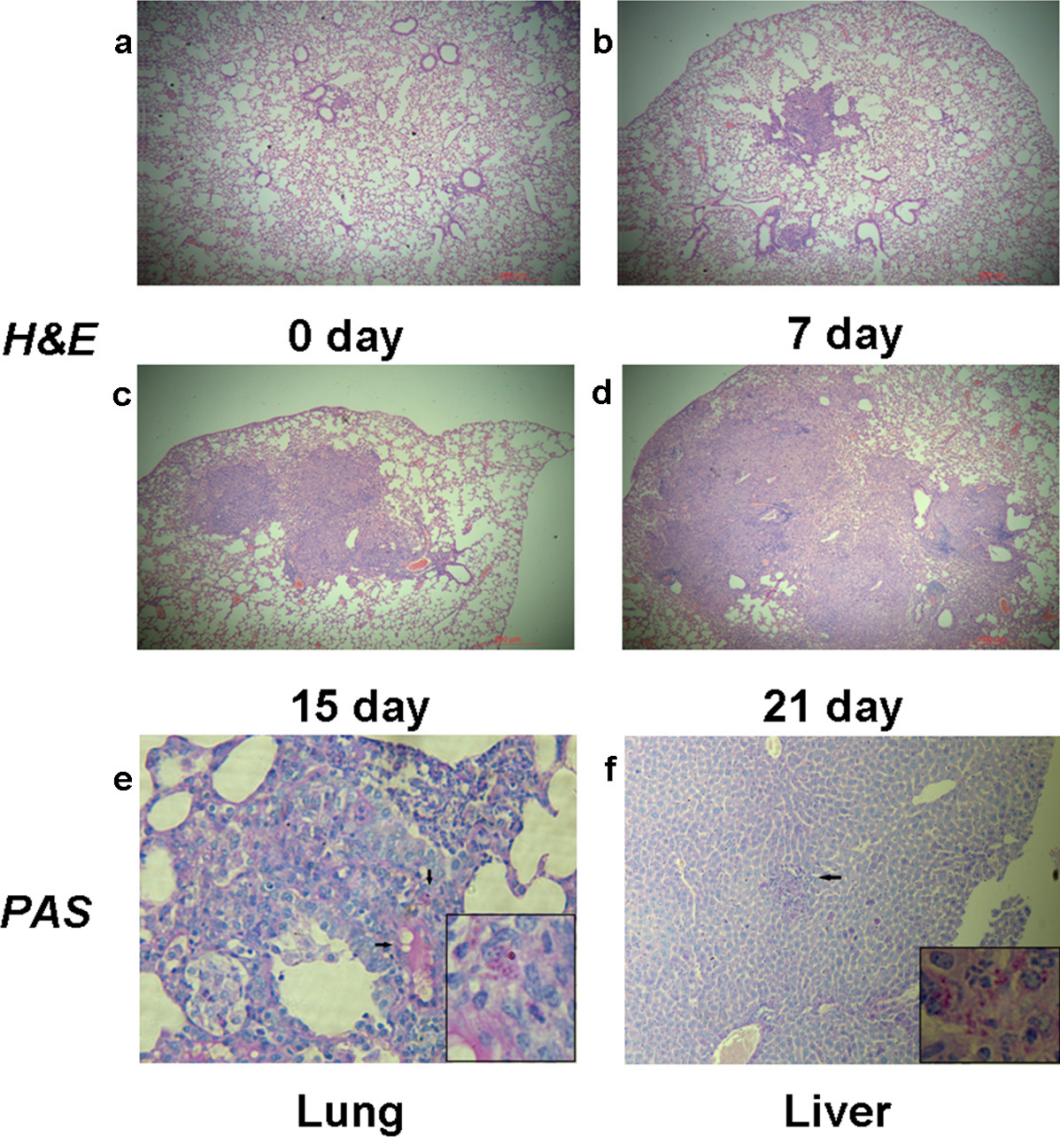
Lung sections of mice infected with *T*. *marneffei*. Progressive inflammatory reaction presented with leukocyte infiltration and granuloma formation with tissue destruction, HE-stains 100X (a-d). Macrophages loaded with yeast cells and formation of multinucleated giant cells in lung 7 days post infection, PAS stains 400X, partial enlarged panel (1000X) depicts fission yeast (e). Macrophages loaded with yeast cells in liver 11 days post infection (f).

Aerosolizing 8 mL of the suspension containing 10^8^/mL conidia for 60 min resulted in lethal infection with mortality of 65% in 40 days (Fig 2A). Death most often occurred during late infection, from the fifteenth day after inoculation. We counted the colonies of *T*. *marneffei* that were present in the lung, liver, spleen and kidney tissues at 3 days, 7 days, 15 days, 21 days and 30 days after inhalation of conidia. Differences between the numbers of CFU in lungs versus livers, spleens or kidneys were significant (*P* = 0.0087, 0.0045, 0.0011 respectively) (Fig 2B). We also found that *T*. *marneffei* was confined to lung tissues until the seventh day after infection, and then diffused to remote organs. We speculated that the liver might be the first infected remote organ followed by spleen and kidney. This speculation is based on the observation that we can’t find fungal infection evidence in other organs except for the lung and the liver in the mice. Histopathological examination by H&E and PAS staining showed inflammatory cells infiltration and fungal burden in these tissues. At day 7, large numbers of macrophages loaded with yeast cells and multinucleated giant cells were identified in lung tissues (Fig 3E). By day 11, similar pathological changes were observed in liver tissues (Fig 3F). HE staining revealed the complete process of lung inflammation, primarily characterized by leukocyte infiltration and granuloma formation with tissue destruction caused by robust fungal invasion (Fig 3A–3D).

### Specificity and Sensitivity of nested PCR

**Fig 4:**
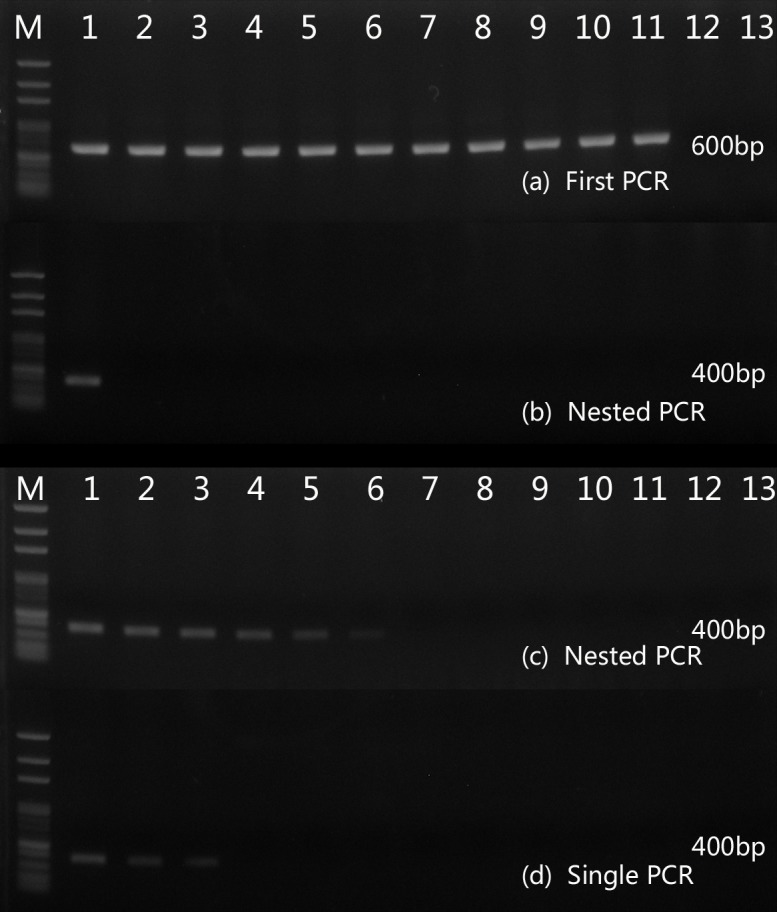
Specificity and sensibility of nested PCR. A 600-bp PCR product were amplified from all fungal samples (a) and a 400-bp PCR product was amplified from *T*. *marneffei* samples (b). M, 100-bp-ladder DNA; Lane1 to 13, *T*. *marneffei*, *Aspergillus flavus*, *Aspergillus fumigatus*, *Aspergillus niger*, *Cryptococcus neoformans*, *Candida albicans*, *Candida krusei*, *Fonsecaea pedrosoi*, *Fonsecaea monophora*, *Histoplasma capsulatum*, *Paracoccidioides brasiliensis*, Negative control (mice DNA), Negative control (water) respectively. Sensitivity of nested PCR (c) and single PCR (d) was 8.4×10^4^ and 8.4×10^7^ fg/μl, respectively. M, 100-bp-ladder DNA; lanes 1 to 13, 8.4×10^9^, 8.4×10^8^, 8.4×10^7^, 8.4×10^6^, 8.4×10^5^, 8.4×10^4^, 8.4×10^3^, 8.4×10^2^, 8.4×10^1^, 8.4×10^0^, 8.4×10^−1^, 8.4×10^−2^, 8.4×10^−3^ f g /μl, respectively.

To determine the specificity of nested PCR, DNA from selected fungal species and mice were analyzed. Primers RRF1 and RRH1 specific to fungi result in approximately 600-bp PCR products in all fungal samples (Fig 4A). Primers pm1 and pm2 specific to *T*. *marneffei* produce approximately 400-bp PCR products in *T*. *marneffei* samples. While mice and other fungal control samples gave negative PCR results (Fig 4B). Sensitivity of the nested PCR with primer pairs RRF1 and RRH1 and Pm1 and Pm2 and single PCR with specific primers Pm1 and Pm2 was 8.4×10^4^ and 8.4×10^7^ fg/μl, respectively (Fig 4C and 4D).

### Detection of T. marneffei in fresh tissue and BALF by Nested PCR

**Fig 5:**
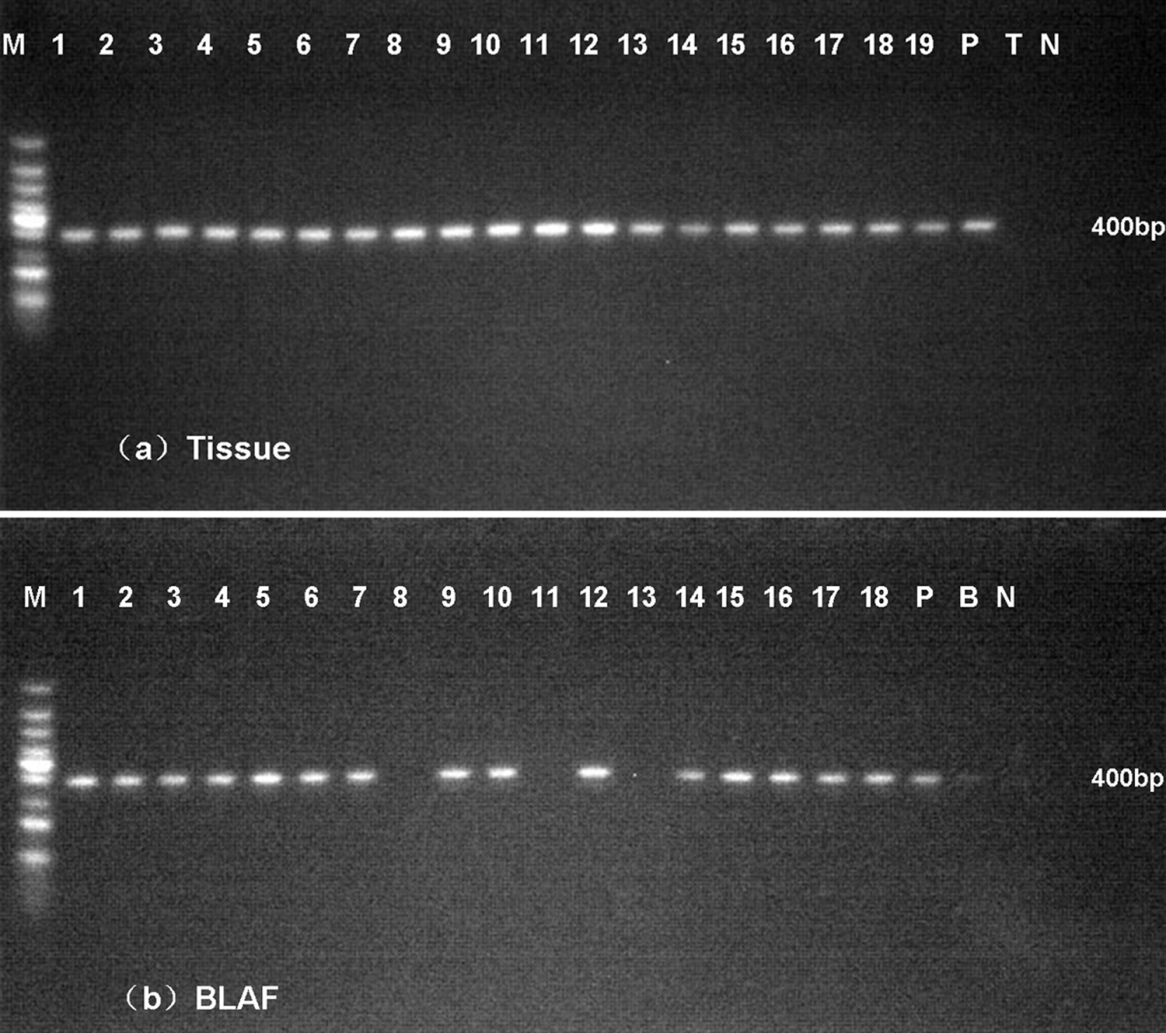
**Nested PCR assays in fresh lung tissues (a) and BALF (b).** A 400-bp specific product was amplified in most of samples. M: 100-bp-ladder DNA, P, purified genomic DNA from *T*. *marneffei*, T, lung tissue from healthy mice, B, BALF from healthy mice, N, water.

A total of 19 samples of lung tissues and 18 samples of BALF were obtained from 19 infected mice confirmed by CFU assay. Sterile water, BALF and fresh tissues from healthy mice were used as the negative control. Purified genomic DNA from *T*. *marneffei* was used as the positive control. We performed nested PCR in all these samples and detected a 400-bp product by agarose gel electrophoresis (Fig 5).

## Discussion

Murine models are critical for studying the pathogenesis and diagnosis of clinically important fungi. In this study, we first constructed a murine pulmonary model that would mimic human penicilliosis marneffei utilizing inhalation exposure. We found that a nested PCR assay employed in this model might contribute to the development of new diagnostic methods in detecting *T*. *marneffei* in fresh tissues.

Infection with penicilliosis marneffei begins by inhalation of the infectious conidia of *T*. *marneffei*. Thus, the pulmonary route of infection would be optimal to initiate the model, for it can closely mimic the natural route of infection into human. Recently, Buskirk et al. developed a nose-only, acoustical generator exposure system to aerosolize fungal conidia to recapitulate human exposures, but this system was unavailable for most of laboratory in developing country due to the high cost [17]. In this study, we adapted a new inhalation apparatus, which is relatively simple, low cost and timesaving. It could deliver fungal conidia directly to the alveoli of mice, and allow for infection of up to 30 mice simultaneously. The relatively small infectious inoculum (5×10^3^) could result in infection with long duration of 40 days, which provided a reasonable window for further study of the disease. Survival and CFU assay revealed that invasive pulmonary infection occurred in about 65% of infected mice. CFU assay also showed that the lung appears to be the predominant organ during infection and the liver might be the first infected remote organ, consistent with the findings in our previous study [7]. Furthermore, we observed similar histopathological changes of infected mice with human penicilliosis marneffei by using this model. The progressive lung inflammation displayed in this model was consistent with the histopathological findings of reported cases of penicilliosis marneffei [18]. Our results indicated that the model could faithfully simulate human infection of penicilliosis marneffei.

In addition, our study revealed that nested PCR had high specificity and sensitivity in detecting *T*. *marneffei* in fresh tissues and BALF. Till now, diagnosis of fungal infections has been a significant challenge, although nested PCR assays have been reported as powerful tools for detecting pathogenic fungi [9, 12]. To our knowledge, it was previously used to identify *T*. *marneffei* in paraffin embedded tissue and whole blood samples [9, 12]. This is the first time that nested PCR has been used for the detection of *T*. *marneffei* from fresh tissues and BALF and proved to be suitable for detecting *T*. *marneffei*. Compared with CFU results, we found equivalence between nested PCR and CFU in fresh tissues, but two samples revealed negative results in BALF. The discrepancies might be explained by the aforementioned, unsuccessful DNA extraction. These results prompted us to hypothesize that BALF can’t entirely replace lung tissues to reflect pulmonary infection. While in the clinic, lung biopsies are not routinely performed because this invasive examination may make patient’s respiratory function worse. BALF could be obtained by non-invasive procedure, most likely proving favorable for patients. According to the results, we could conclude that nested PCR assay on BALF has high predictive value in reflecting pulmonary infection with a positive rate of 83.3%.

In summary, we successfully developed a novel inhalation exposure system to initiate the model by directly delivering conidia to alveoli, and verified that nested PCR on BALF may serve as a good alternative tool to conventional diagnosis of penicilliosis marneffei. This simple model provides an alternative to existing intranasal models and may contribute to deeper insight into penicilliosis marneffei.
